# Marginal structural Cox model for estimating the effect of Chinese medicine on the survival of people living with HIV: a 17-year real-world retrospective cohort study

**DOI:** 10.3389/fpubh.2025.1722889

**Published:** 2025-12-12

**Authors:** Wanqi Pan, Qianlei Xu, Yanmin Ma, Liran Xu, Huangchao Jia, Dongli Wang, Keying Zhu, Miao Zhang, Juan Wang, Huijun Guo, Yantao Jin

**Affiliations:** 1 The First Clinical Medical School, Henan University of Chinese Medicine, Zhengzhou, China; 2 Department of Acquired Immune Deficiency Syndrome Treatment and Research Center, First Affiliated Hospital of Henan University of Chinese Medicine, Zhengzhou, China; 3 Center for AIDS/STD Control and Prevention, Center for Disease Control and Prevention of Henan Province, Zhengzhou, China; 4 Henan Key Laboratory of Viral Diseases Prevention and Treatment of Chinese Medicine, Henan University of Chinese Medicine, Zhengzhou, China

**Keywords:** retrospective cohort study, human immunodeficiency virus, acquired immune deficiency syndrome, marginal structural Cox model, Chinese medicine, survival

## Abstract

**Objective:**

The aim of this study was to evaluate the long-term effect of Chinese medicine (CM) on the survival of people living with HIV (PLHIV) by investigating the potential synergistic effects of combining CM with antiretroviral therapy (ART).

**Methods:**

We conducted a 17-year cohort study based on standardized case registry data, using various packages in the R software. PLHIV enrolled in the national CM HIV treatment trial were classified as the CM group, while PLHIV not enrolled were classified as the Non-CM group. CD4+T cell count and ART regimen were collected annually during the period from the cohort start to the endpoint. The cumulative observations of person-years and mortality were computed using life table analysis. The cumulative survival rates and survival curves were compared using Kaplan-Meier and log-rank tests. CD4+T cell count and ART regimen were time-dependent covariates, and a marginal structural Cox model was used to control for these variables in evaluating the effect of CM on the survival of PLHIV.

**Results:**

A total of 2,924 PLHIV were included in the analysis, comprising 1,210 in the CM group and 1,714 in the Non-CM group. The mortality was significantly lower in the CM group than that in the Non-CM group (2.92/100 person-years vs. 3.86/100 person-years, *P* < 0.001). Multivariate analysis showed that the risk of death in the CM group was 0.86 compared with the Non-CM group (95% CI: 0.77–0.97, *P* < 0.05). The mortality risk of patients who received first-line ART and second-line ART was 0.47 (95% CI: 0.39–0.56, *P* < 0.05) and 0.49 (95% CI: 0.38–0.62, *P* < 0.001), respectively, compared with those who did not receive ART.

**Conclusion:**

The results demonstrate that after controlling the time-dependent covariates with a marginal structural Cox model, CM could improve the long-term survival rate of PLHIV by investigating the potential synergistic effects of combining CM with ART.

## 1 Introduction

Acquired immune deficiency syndrome (AIDS) is induced by human immunodeficiency virus (HIV) and continues to be a significant global public health problem1 (1, 2). Since the beginning of the AIDS epidemic, a total of 40.1 million people have died of AIDS-related illnesses. In 2021, there were an estimated 38.4 million people living with HIV (PLHIV) globally, 1.5 million people newly infected with HIV, and 650 thousand people who died of AIDS-related illness (3). Antiretroviral therapy (ART) is the main treatment for PLHIV, which can significantly extend the life expectancy of PLHIV. This is a consensus in the field of HIV clinical epidemiology. Although ART has resulted in a dramatic reduction in mortality among PLHIV and the life expectancy between PLHIV and the general population has narrowed (4–6), the life expectancy of PLHIV is still 8 years shorter than that of the general population (7). Furthermore, PLHIV with a low CD4+T cell count at the start of ART have an even larger gap in life expectancy (8). In China, Chinese medicine (CM) plays an important role in the treatment of infectious diseases, including AIDS. It has been demonstrated that CM increases the CD4+T cell count (9), promotes immune reconstitution (10, 11), reduces adverse reactions to ART (12), and improves the symptoms of PLHIV. Therefore, it is necessary to evaluate the therapeutic effect of CM in improving the survival rate of PLHIV.

Many studies have suggested that CM could reduce the risk of death and prolong the survival time of PLHIV (13–17). As an important therapy widely used in the course of HIV/AIDS, ART is an important covariate when evaluating the efficacy of CM. However, previous studies only analyzed the baseline ART, while failing to consider the ART as a function of the change of the covariate (13, 14). In addition, as a repeated measurement variable, ART varies over time and becomes a time-dependent variable due to medication compliance or scheme adjustment due to resistance and adverse effects. Since April 2005, China has revised the free HIV/AIDS ART program four times, and first-line ART for PLHIV is adjusted constantly to second-line ART. Additionally, ART not only influences mortality, but also affects the CD4+T cell count (18–20). Thus, ART has also become a time-dependent confounding factor during the 17-year follow-up period. The standard approaches to estimate of the effect of CM, such as regression analysis (21), may be biased when time-varying covariance has occurred (22). The marginal structural Cox model (MSCM) can control the bias of time-dependent covariates and has been widely used in recent years (23, 24) and is a more suitable method to study the survival impact of PLHIV with time-varying covariates.

In 2004, the State Administration of Traditional Chinese Medicine of China initiated a National CM HIV/AIDS Treatment Trial Program (NCMTP) to treat PLHIV, and Henan is one of the earliest provinces to start NCMTP. Individual who participated in the NCMTP were voluntary and signed the informed consent. All patients who participated in the NCMTP of Henan Province were given the free CM preparation “Yiaikang capsule” (produced by the preparation room of Henan Provincial Institute of Chinese Medicine with pharmaceutical products approval number: LZ05002, containing substances such as Ginseng, Astragalus, Atractylodes Rhizome, Fragrant Solomon's Seal Rhizome, Angelica sinensis, Szechwan Lovage Rhizome, White Peony Root, and Baical Skullcap Root), and ingesting five capsules three times daily (14). Additional CM treatment was given according to the individual patient's symptoms. The details of the NCMTP have been reported elsewhere (13, 25). In order to evaluate the long-term effect of CM on the survival of PLHIV by investigating the potential synergistic effects of combining CM with ART, a 17-year real-world study was conducted based on register of NCMTP and the National Free Antiretroviral Treatment Program (NFATP), and the MSCM was used to control the bias of time-dependent covariates.

## 2 Methods

### 2.1 Study population

This retrospective cohort study was conduct in cities of Kaifeng, Zhoukou, Zhumadian, Nanyang and Shangqiu in Henan province in China, where PLHIV could enroll in the NCMTP before October 2004. Patients were eligible for inclusion if they met the following criteria: confirmed with HIV infection between October 2000 and October 2004 with a Western blot result, aged 18–60 years, follow-up period of longer than 1 month, and with complete baseline information. The individuals with enrollment in the NCMTP were assigned to the CM group, and others in the same area were taken as Non-CM group. Patients who participate in the NCMTP after October 2004 were excluded.

### 2.2 Data collection and variables

The data analyzed in this study were collected from standard medical record registers NFATP and NCMTP of Henan Province. The NFATP and NCMTP were conducted by different departments with mutual non-interference. The collected data included demographic characteristics, such as gender, date of birth, marital status, occupation, educational level, transmission route, HIV positive confirmation time, time of participation and withdrawal from the NCMTP, and time of death. All information about time of taking ART and the regimen, as well as CD4+T cell count and time, which was recorded in the medical record registers, were collected. Except for the information of time of participation and withdrawal from the NCMTP which were obtained from the register of the NCMTP, all of other information were obtained from the register of the NFATP. Meanwhile, the CD4+T cell count records were obtained from the both registers.

The start of the cohort was 1st October 2004 and the primary endpoint was all-cause death. CD4+T cell count and ART regimen were collected annually during the period from the cohort start to the endpoint. Individuals who were alive after 1st October 2021, lost to follow-up, or withdrew from treatment with CM or ART were taken as censored data. The ART regimen comprising lopinavir/ritonavir was taken as second-line ART, while other regimens were considered first-line ART.

### 2.3 Data analysis

The latest CD4+T cell count recorded within 6 months of the annual anniversary date of cohort commencement was used as the CD4+T cell count in the study analysis; if no value was entered during this period, the variable was defined as missing. Continuous variables were presented as means and standard deviations, and comparisons between groups were made using the Student's *t*-test. Categorical variables were summarized as frequencies and proportions and were compared between groups using the chi-squared test. The cumulative observations of person-years and mortality were computed using life table analysis. The cumulative survival rates and survival curves were compared using Kaplan–Meier and log-rank tests.

Age, administration of ART, and CD4+T cell count were time-dependent covariates, while group, gender, marital status, education level, transmission route, and HIV positive confirmation time were normal covariates. The MSCM was used to examine the association of CM with the risk of death among PLHIV with adjustments for time-dependent and normal confounders, and expressed as hazard ratio (HR) and 95% confidence interval (CI).

All data analyses were performed using R (version 4.0.1), with two-sided *P*-values < 0.05 considered indicative of statistical significance. The following R packages were used in the analyses: data.table, plyr, lubridate, compareGroups, ggplot2, survival, survminer, tibble, etc.

### 2.4 Ethical considerations

This study was approved by the Institutional Review Board of the First Affiliated Hospital of Henan University of Chinese Medicine (2019HL-068). Individual informed consent was not obtained because this study analyzed existing data collected during the course of routine treatment, and the data were reported in aggregate without the use of individual identifying information.

## 3 Results

### 3.1 Patients

A total of 2,924 PLHIV met the criteria for inclusion and were enrolled in the study. Among them, 1,210 (41.38%) were in the CM group and 1,714 (58.62%) were in the Non-CM group. There were 1,445 (49.42%) males and 1,479 (50.58%) females. Most participants were married (74.59%). There were 1,802 (61.63%) participants who had undergone less than 6 years of education. A total of 2,910 (99.52%) were farmers, and 2,759 (94.36%) were infected with HIV through plasma. Almost all (99.79%) belong to the Han nationality. The proportion of PLHIV who had not received ART and who had taken ART at baseline was 52.60 and 47.40%, respectively. The characteristics of the CM group and Non-CM group are shown in Table 1.

**Table 1:** Characteristics of the study population at baseline.

**Characteristic**	**Overall**	**Non-CM**	**CM**	***P-*value**
**Gender**				0.795
Male	1,445 (49.42%)	851 (49.65%)	594 (49.09%)	
Female	1,479 (50.58%)	863 (50.35%)	616 (50.91%)	
**Age (years)**				0.728
< 40	1,473 (50.38%)	871 (50.82%)	602 (49.75%)	
40–50	1,047 (35.81%)	613 (35.76%)	434 (35.87%)	
>50	404 (13.81%)	230 (13.42%)	174 (14.38%)	
**Marital status**				0.728
Single/widow(er)	743 (25.41%)	431 (25.15%)	312 (25.79%)	
Married	2,181 (74.59%)	1,283 (74.85%)	898 (74.21%)	
**Ethnicity**				0.411
Han	2,918 (99.79%)	1,709 (99.71%)	1,209 (99.92%)	
Others	6 (0.21%)	5 (0.29%)	1 (0.08%)	
**Occupation**				0.073
Farmer	2,910 (99.52%)	1,702 (99.30%)	1,208 (99.83%)	
Others	14 (0.48%)	12 (0.70%)	2 (0.17%)	
**Education (years)**				0.805
≤ 6	1,802 (61.63%)	1,060 (61.84%)	742 (61.32%)	
>6	1,122 (38.37%)	654 (38.16%)	468 (38.68%)	
**Transmission route**				< 0.001
Plasma	2,759 (94.36%)	1,576 (91.95%)	1,183 (97.77%)	
Others	165 (5.64%)	138 (8.05%)	27 (2.23%)	
**Treatment with ART**				< 0.001
No	1,538 (52.60%)	966 (56.36%)	572 (47.27%)	
Yes	1,386 (47.40%)	748 (43.64%)	638 (52.73%)	
**CD4**+**T cell count (cells/**μ**l)**				< 0.001
< 200	988 (33.79%)	729 (42.53%)	259 (21.41%)	
200–350	747 (25.55%)	497 (29.00%)	250 (20.65%)	
351–500	557 (19.05%)	282 (16.45%)	275 (22.73%)	
>500	632 (21.61%)	206 (12.02%)	426 (35.21%)	

### 3.2 PLHIV with ART by years

**Figure 1:**
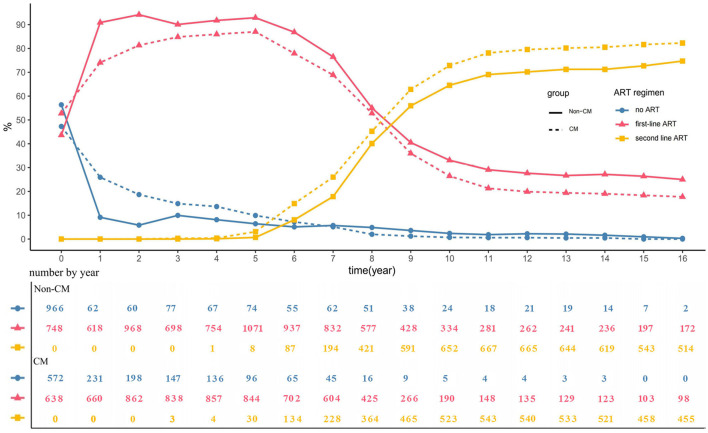
Comparison of ART percentage between the two groups of PLHIV.

The proportion of PLHIV who had not received ART decreased over time in both groups. The proportion of PLHIV who had not received ART in the Non-CM group was 9.12% at 1 year, 6.42% at 5 years, 2.38% at 10 years, 0.94% at 15 years, and 0.29% at 16 years. The proportion of PLHIV who had not received ART in the CM group was 25.93% at 1 year, 9.90% at 5 years, 0.70% at 10 years, 0.00% at 15 years, and 0.00% at 16 years. Among PLHIV taking ART, the proportion taking first-line ART decreased over time, while the proportion taking second-line ART increased. The details of these data are shown in Figure 1.

### 3.3 Mortality and cumulative survival

In the Non-CM group, 1,714 patients were followed up for 20,374 person-years, during which 787 (45.92%) died and 211 (12.31%) were lost to follow-up. The mortality was 3.86/100 person-years; 85.94% of patients were alive at 1 year, 71.95% were alive at 5 years, 63.20% were alive at 10 years, 55.31% were alive at 15 years, and 52.91% were alive at 17 years.

**Figure 2:**
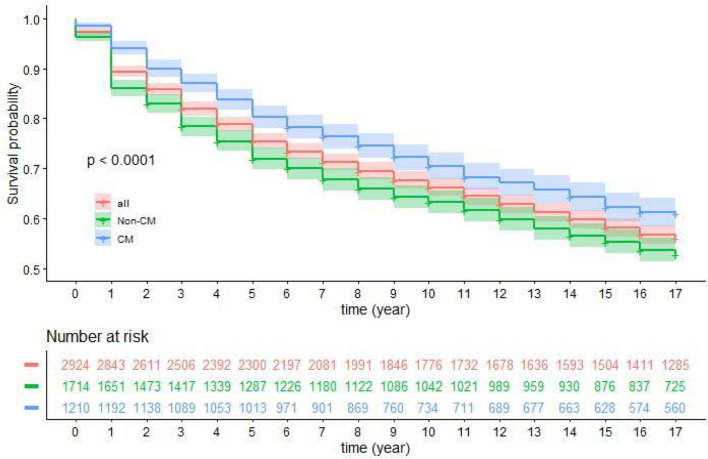
Cumulative survival in the two groups of PLHIV.

In the CM group, 1,210 patients were followed up for 15,106 person-years, during which 441 (36.47%) died and 213 (17.60%) were lost to follow-up. The mortality was 2.92/100 person-years; 94.05% of patients were alive at 1 year, 80.25% were alive at 5 years, 70.38% were alive at 10 years, 62.30% were alive at 15 years, and 60.99% were alive at 17 years. The cumulative survival significantly differed between the two groups (Figure 2).

### 3.4 Effect of CM on survival among PLHIV by MSCM

After multivariable adjustment in the MSCM, the adjusted hazard ratio (AHR) for mortality in the CM group was 0.86 (95% CI, 0.77–0.97) compared with the Non-CM group. Compared with PLHIV who did not receive ART, the AHRs for mortality in the groups who received first-line ART and second-line ART were 0.47 (95% CI, 0.39–0.56) and 0.49 (95% CI, 0.38–0.62), respectively. Compared with participants with a baseline CD4+T cell count of < 200 cells/μl, the mortality of participants with a CD4+T cell count of 200–350, 351–500, and >500 cells/μl were 0.29 (95% CI, 0.25–0.34), 0.2 (95% CI, 0.16–0.24), and 0.15 (95% CI, 0.12–0.18), respectively. Table 2 summarizes the HR estimated by the MSCM.

**Table 2:** Hazard ratios for variables affecting AIDS-related death of PLHIV by MSCM.

**Variables**	**Unadjusted**			**Adjusted**		
	**HR**	**95%CI**	***P*** **value**	**AHR**	**95%CI**	***P*** **value**
**Group**						
Non-CM	1.00	—	—	1.00	—	—
CM	0.75	0.67–0.84	< 0.001	0.86	0.77–0.97	0.014
**ART**						
No	1.00	—	—	1.00	—	—
First-line	0.61	0.52–0.72	< 0.01	0.47	0.39–0.56	< 0.001
Second-line ART	0.79	0.62–0.99	0.045	0.49	0.38–0.62	< 0.001
**CD4**+**T cell count (cells/**μ**l)**						
< 200	1.00	—	—	1.00	—	—
200–350	0.30	0.26–0.34	< 0.001	0.29	0.25–0.34	< 0.001
351–500	0.20	0.17–0.24	< 0.001	0.20	0.16–0.24	< 0.001
>500	0.15	0.12–0.18	< 0.001	0.15	0.12–0.18	< 0.001
**Gender**						
Male	1.00	—	—	1.00	—	—
Female	0.69	0.61–0.77	< 0.001	0.68	0.60–0.76	< 0.001
**Age (years)**						
< 40	1.00	—	—	1.00	—	—
40–50	1.00	0.85–1.17	0.969	0.98	0.83–1.15	0.813
>50	1.70	1.46–1.99	< 0.001	1.71	1.45–2.02	< 0.001
**Education (years)**						
≤ 6 years	1.00	—	—	1.00	—	—
>6 years	0.78	0.69–0.87	< 0.001	0.81	0.71–0.91	0.001
**Marital status**						
Single/widow(er)	1.00	—	—	1.00	—	—
Married	1.09	0.96–1.23	0.191	1.22	1.08–1.39	0.002

PLHIV, people living with human immunodeficiency virus; MSCM, marginal structural Cox model; CM, Chinese medicine; ART, antiretroviral therapy; HR, hazard ratio; CI, confidence interval.

## 4 Discussion

ART plays an irreplaceable role in the treatment of PLHIV, having transformed HIV from a fatal disease into a manageable chronic illness. At the same time, a number of studies have shown that CM prolongs the survival time of PLHIV (26, 27). However, previous studies failed to consider ART as a time-dependent covariate when evaluating the efficacy of CM, which may lead to bias. In the present study, we used the MSCM to control the time-dependent covariates. To the best of our knowledge, this is the first study using MSCM to evaluate the long-term protective effect of CM on the survival of PLHIV by investigating the potential synergistic effects of combining CM with ART in a longitudinal cohort of PLHIV.

In the present study, the mortality was 2.92/100 person-years in the CM group and 3.86/100 person-years in the Non-CM group, which shows that the mortality risk of PLHIV in the CM group was lower than that of PLHIV in the Non-CM group. A study conducted in China followed 1,666 PLHIV for 102,591 person-months and showed that the total mortality of PLHIV was 3.6/100 person-years after CM therapy (28), which was lower than the worldwide mortality of PLHIV. Another study reported that the mortality of PLHIV was 2.97/100 person-years in the CM group and 3.79/100 person-years in the Non-CM group (13). These studies all show that CM reduces the mortality of PLHIV. In addition, after multivariable adjustment in the MSCM, the present study showed that the 17-year cumulative survival rate of PLHIV who received CM therapy was significantly higher than that of PLHIV without CM (AHR 0.86, 95% CI: 0.77–0.97, *P* < 0.05). Therefore, the present findings also suggest that CM decreases the mortality of PLHIV. In CM, the pathology guides the clinical practice. In our view, the core pathogenesis of AIDS is spleen qi deficiency. After entering the human body, HIV first damages the spleen. The spleen is the root of acquired constitution and the source of qi and blood generation, and its most fundamental and important function is transformation of water and food. Once the spleen is damaged, qi, blood, and the five zang-organs will lose nourishment and become weak (29, 30). The CM Yiaikang used in the CM group could invigorate the spleen and supplement qi, nourish yin and blood, dispel wind, and clear heat (31). In clinical use, it could effectively delay the progress of the disease, and reduce mortality (13). In the molecular mechanism studies, Yiaikang has shown strong inhibitory effects on viral replication and immune-boosting properties, which are achieved through inhibition of the viral transactivator of transcription (Tat) and regulatory protein Rev, and host intercellular adhesion molecule 1 (ICAM-1) (32).

Many studies have examined the association between ART and mortality events, and there is broad consensus that ART reduces the mortality of PLHIV (33–35). However, these studies mostly evaluated first-line or second-line ART, and only considered the baseline values. The present study analyzed the impact of a dynamic ART regimen on the mortality of PLHIV, which is more in line with the real situation. Using the MSCM, the mortality risk of patients receiving first-line ART and second-line ART was 0.47 (95% CI: 0.39–0.56, *P* < 0.001) and 0.49 (95% CI: 0.38–0.62, *P* < 0.001), respectively, compared with patients who did not receive ART. This result indicates that both first-line and second-line ART had a significant effect in reducing the death risk of HIV/AIDS, which is consistent with many recent studies (36–40). In addition, we found that the risk of death was not significantly different between the patients receiving first-line and the patients receiving second-line ART, which is not consistent with one study reporting that the mortality of PLHIV with second-line ART is lower than that of PLHIV without second-line ART (25). This discrepancy may be explained by the timely use of second-line ART in our study; however, it requires further investigation.

Our study also found that the mortality of PLHIV was associated with the CD4+T cell count, education level, age, gender, and marital status. PLHIV with a higher CD4+T cell count had a lower mortality, which is consistent with many studies (41, 42). The present study also found that the mortality risk was higher in males compared with females, which is in line with previous studies (13, 43, 44). This gender-related difference in mortality may be caused by the differences between males and females in physiological functions and living habits. In addition, the life expectancy also differs between males and females (45), which may also have affected the present results. Age was also a strong predictor of disease progression and mortality risk, as older PLHIV have a poor immune response (46) and are more likely to develop AIDS-related and non-related diseases. Evidence of the effect of age on AIDS-related deaths is best reflected by the lower mortality among adolescents (47) and the higher mortality among older adults (48). Finally, a higher level of education was associated with a significantly lower mortality among PLHIV, probably because educated PLHIV have a higher level of knowledge about HIV/AIDS.

Our study has several strengths. First, we used the MSCM, which control the influence of time-dependent covariates on the results. Second, the follow-up time was nearly 20 years, which enabled us to objectively analyze the factors affecting the survival of PLHIV. However, our study also has several limitations. First, as a real-world study, selection bias was not inevitable. Meanwhile, the important factor affecting the evaluation of therapeutic efficacy, such as medication adherence, cannot be obtained. Second, since this study was based on a routine database that lacked information on many variables, some risk factors associated with the mortality of PLWH such as the viral load, comorbidity of AIDS like related malignancies, cardiovascular events, renal and hepatic diseases, and neurocognitive diseases (49) were not recorded in the medical registers in this study and thus could not be analyzed. Finally, most patients included in this study were infected through the blood, which may limit the generalizability of results. Therefore, prospective studies with more rigorous designs should be carried out to confirm our primary results.

## Data Availability

The raw data supporting the conclusions of this article will be made available by the authors, without undue reservation.
